# Percutaneous coronary intervention for congenital absence of the right coronary artery with acute myocardial infarction: A case report and literature review

**DOI:** 10.1097/MD.0000000000018981

**Published:** 2020-01-31

**Authors:** Wei-Chao Liu, Qiang Qi, Wei Geng, Xiang Tian

**Affiliations:** Baoding No. 1 Central Hospital, Baoding, China.

**Keywords:** acute myocardial infarction, computed tomography angiography, percutaneous coronary intervention, right coronary artery absence

## Abstract

**Rationale:**

Congenital absence of the right coronary artery with acute myocardial infarction (AMI) is a rare clinical situation that may lead to death. We report a case of successful percutaneous coronary intervention for congenital absence of the right coronary artery with AMI.

**Patient concerns:**

A 53-year-old woman had a 7-day history of chest discomfort that had worsened over 10 hours. She was diagnosed as having myocardial infarction and was admitted to hospital.

**Diagnosis:**

Coronary angiography showed absence of the right coronary artery; the left anterior descending (LAD) branch sent out the right ventricular branch and the posterior descending branch. The LAD branch was occluded and there was diffuse stenosis of the middle right ventricular branch and severe stenosis of the distal circumflex branch.

**Interventions:**

Percutaneous coronary intervention was performed. One stent was implanted in the LAD branch and another implanted in the right ventricular branch.

**Outcomes:**

The patient was discharged 3 weeks after surgery. The follow-up showed that the patient was asymptomatic without recurrence.

**Lessons:**

Although absence of the right coronary artery with AMI is a fatal condition, percutaneous coronary intervention remains an effective treatment.

## 1 Introduction

Congenital absence of the right coronary artery (RCA) is a rare case of coronary artery malformation; it is even more rarely associated with acute coronary syndrome or acute myocardial infarction (AMI), but this association may lead to death.^[1–3]^ It has been speculated that the reason for the congenital absence of the RCA is that it does not develop during the embryonic period, or because congenital occlusion of the RCA occurs with or without other congenital heart diseases.^[4]^

In this study we report a case of successful percutaneous coronary intervention (PCI) for congenital absence of the RCA with AMI. One month after PCI, coronary artery computed tomography angiography (CTA) was performed to confirm absence of the RCA. This study facilitates understanding of the management of congenital absence of the RCA with AMI, which has rarely been reported previously.

## 2 Case report

A 53-year-old woman was admitted to our hospital with the chief complaint of paroxysmal chest tightness lasting for 7 days, which had become aggravated over the previous 10 hours. Seven days before admission, the patient suffered from chest tightness during sleep, which lasted about 2 hours and could be relieved by changing to the sitting position. The patient did not go to the hospital. The chest tightness recurred 10 hours before admission and persisted without relief; it was accompanied by palpitation, dizziness, and cough. The patient's symptoms gradually worsened, prompting her to come to our hospital for treatment.

The patient had a history of hypertension and diabetes of 3 years. She regularly took antihypertensive and hypoglycemic drugs without monitoring blood pressure and blood sugar. On physical examination, the patient's temperature was 36.6°C, pulse rate was 94/min, respiratory rate was 16/min, and blood pressure was 144/98 mm Hg. The breathing sounds of both lungs were rough, and moist rales could be heard in both lungs. Cardiac examination findings were normal. Electrocardiogram (ECG) revealed sinus rhythm, and I, aVL, and V1 to V5 ST segment elevation. Creatine kinase isoenzyme (CK-MB) levels were 5.9 ng/mL (reference range, 0–4.3 ng/mL), myoglobin (MYO) levels were 315 ng/mL (reference range, 0–107 ng/mL), Troponin I (cTNI) was 4.4 ng/mL (reference range, 0–0.4 ng/mL), brain natriuretic peptide (BNP) was 1510 pg/mL (reference range, 0–100 ng/mL), D-Dimer was <100 ng/mL (reference range, 0–600 ng/mL). A diagnosis of acute extensive anterior and high lateral ST-elevation myocardial infarction was made.

In view of acute heart failure and the high risk of PCI, the patient was transferred to a cardiac intensive care unit (CCU) for treatment. The patient was treated with aspirin (100 mg, qd), clopidogrel (75 mg, qd), atorvastatin (20 mg, qn), isosorbide nitrate (10 mg, Tid), low molecular weight heparin (5000 IU, Q12H), and recombinant human BNP (0.0075 μg/kg/min).

**Figure 1:**
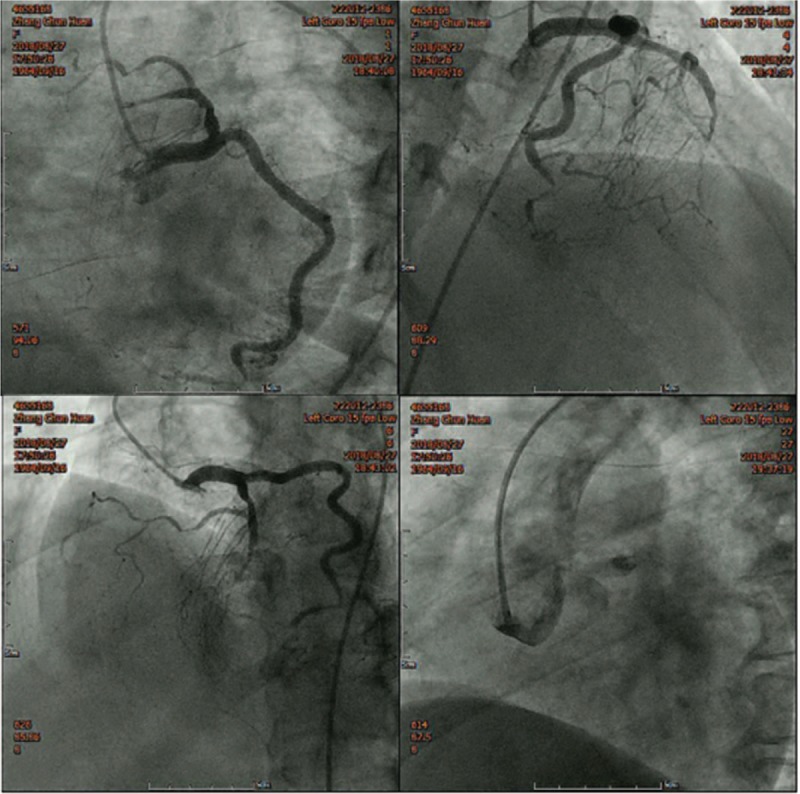
The coronary angiography showed that the mid-distal LAD branch was completely occluded. The RCA was not visible from its origin at the aortic root. The LAD sent out the right ventricular branch and the posterior descending branch. The middle segment of the right ventricular branch was diffusely occluded, up to 95%. LAD = left anterior descending, RCA = right coronary artery.

Ten days later, the patient's condition had improved. BNP dropped to 427 pg/mL. The echocardiography showed segmental wall motion abnormalities, low-limit of normal left ventricular systolic function, and left ventricular ejection fraction of 50% (by the Simpson method). Coronary angiography (CAG) was performed. CAG showed that the mid-distal left anterior descending (LAD) branch was completely occluded. The distal left circumflex (LCX) branch was severely stenosed. The right coronary artery (RCA) was not visible from its origin at the aortic root. The LAD sent out the right ventricular branch and the posterior descending branch. The middle segment of the right ventricular branch was diffusely occluded, by up to 95% (Fig. 1).

**Figure 2:**
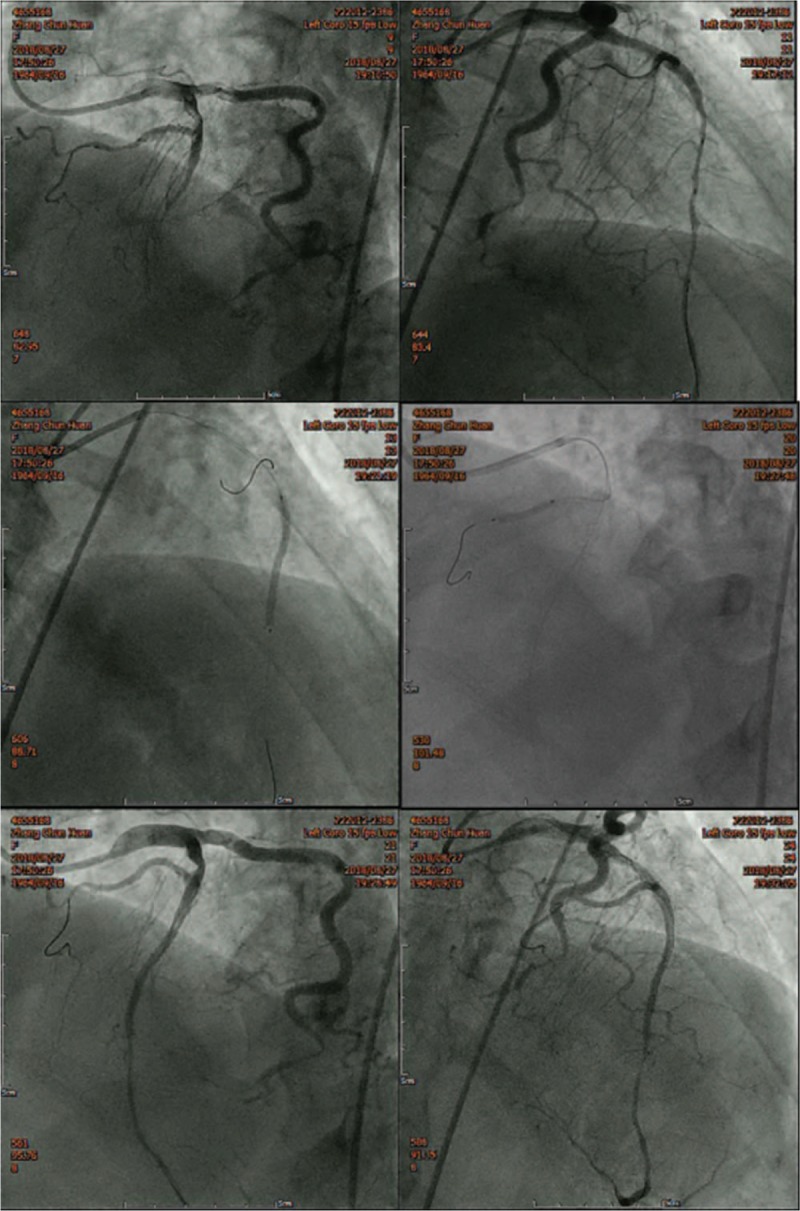
An Excel 2.5 × 36 mm stent was implanted in the stenosed section of the LAD artery. An Excel 2.5 × 14 mm stent was implanted in the stenosed section of the right ventricular branch, which originated from the LAD artery. There was no residual stenosis, no dissection or tear, and thrombolysis in myocardial infarction blood-flow grade was 3. LAD = left anterior descending.

With the consent of the family members, LAD interventional therapy was performed. We inserted a 6-F EBU 4.0 catheter into the left coronary artery orifice; then, a SION guide-wire was sent through the LAD to the right ventricular branch, and a VT guide-wire was sent into the distal segment of the LAD. A Maverick 2.0 × 15 mm balloon was delivered along the SION guide-wire into the narrowed area of the right ventricular branch and was pre-dilatated with 12 atmosphere (atm). Then, the Maverick 2.0 × 15 mm balloon was delivered along the VT guide-wire to the narrowed area of the LAD and pre-dilatated with 12 atm. An Excel 2.5 × 36 mm stent was implanted into the stenosed region of the LAD. Quantum Maverick 3.0 × 12 mm balloon was delivered to the stent and post-dilated with 16 atm. Then, an Excel 2.5 × 14 mm stent was implanted in the stenosed region the right ventricular branch, which originated from the LAD. A Maverick 2.0 × 15 mm balloon was delivered to the stent and post-dilated with 16 atm. There was no residual stenosis, no dissection or tear, and thrombolysis in myocardial infarction blood-flow grade was 3 (Fig. 2).

**Figure 3:**
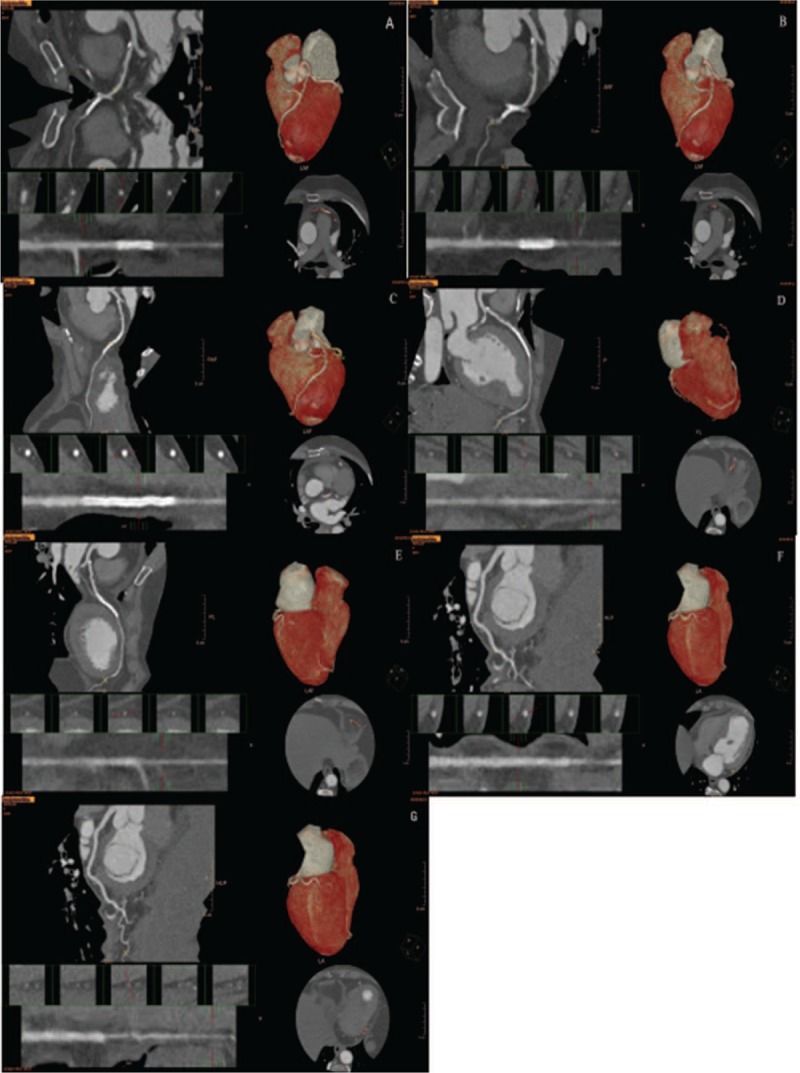
Computed tomography angiography showed that the RCA was not visible from its origin at the aortic root. The RCA originated from the LAD artery. The RCA was short and small with a rough wall. The coronary artery stent could be seen locally, and the corresponding lumen was unobstructed. LAD = left anterior descending, RCA = right coronary artery.

The patient was treated with antiplatelet aggregation, anticoagulation, coronary artery dilation, lipid regulation, and symptomatic treatment after operation. One week later, the patient's symptoms improved, and she was discharged from hospital. Another 2 weeks later, the patient underwent CTA of the coronary artery, which showed that the RCA was not visible from its origin at the aortic root. The RCA originated from the LAD. The RCA was short and small with a rough wall. The coronary artery stent could be seen locally, and the corresponding lumen was unobstructed. The left coronary artery originated from the left sinus orifice. The coronary artery stent could be seen in the middle part of the LAD and the corresponding lumen was unobstructed. The distal LCX was severely stenosed (Fig. 3A–G).

The clinical data used in this study were available in the electronic medical database of Baoding No.1 Central Hospital. This study was approved by the institutional review board of our hospital and the patient provided consent for publication of the case.

## 3 Discussion

### 3.1 Epidemiological study of congenital absence of the RCA

White and Edwards first described the anomalous origin of the RCA, which was a rare congenital anomaly in 1948.^[5]^ With the development of cardiac catheterization, various forms of coronary artery malformation were increasingly found.^[6]^ Lipton et al found that an isolated single coronary artery was a rare congenital anomaly occurring in approximately 0.024% of the population.^[7]^ Ying et al of China used a large sample (22636) of coronary angiographic data from the China Data Analysis found 234 cases of anomalous orifice of the coronary artery, mainly in the RCA (59%). Absence of the RCA (i.e., single left coronary artery) was found in only 5 cases (0.022%).^[8]^ Congenital absence of the RCA or the RCA originating from the LCX is a special type of single coronary artery, and is clinically rare.^[9]^ In the present case, the RCA was congenitally absent, and the LAD sent out the right ventricular branch and the posterior descending branch, which was particularly rare, and has not been reported previously.

### 3.2 Classification of single coronary artery malformations

Angiographic classification of a single coronary artery was first proposed by Lipton in 1979.^[7]^ First, the types of a single coronary artery can be divided into L- and R-types according to the origin of the artery. Second, it can be divided into groups I, II, and III according to the coronary artery anatomical course. Group I represents the extension of the end of a single coronary artery that dominates the area that should be supplied by the contralateral coronary artery. Group II represents a branch divided from the proximity of a single coronary artery that dominates the area that should be supplied by the contralateral coronary artery. In Group III, the LCX and the LAD originate from the trunk of the RCA, respectively. Finally, according to the relationship between the transverse artery and the pulmonary artery and the aorta, there are three types: A, B, and P. A represents the front, B represents the middle, and P represents the back. According to the above classification, absence of the RCA in this case belonged to the L-II type.

According to the possible clinical consequences, Yamanaka et al classified coronary artery abnormalities as benign and potentially dangerous groups.^[10]^ The benign group had no pathological significance. The group with potentially clinical risk included those with a single coronary artery, a coronary artery originating from the pulmonary artery, and left or right coronary arteries originating from the contralateral coronary sinus. Except for the rare types of Lipton L or RII–IIIB, most of these have a good prognosis.^[11]^ In the present case, absence of the RCA belongs to the potentially dangerous group.

### 3.3 Clinical manifestation

Clinical manifestations of congenital absence of the RCA are different. Most patients present with myocardial ischemia symptoms, including angina pectoris, chest tightness, palpitation, atypical chest pain, etc. About half of these patients have left coronary artery branch lesions, and most of them have electrocardiographic manifestations of myocardial ischemia. The remainder of the patients with chest pain do not have left coronary artery disease, and the ECG is mostly negative.^[12]^ Shirani and Roberts believe that only 15% of patients with single coronary artery malformation have angina symptoms, which are associated with their own anatomical deformities.^[13]^ Other studies have shown that the correlation between single coronary atherosclerosis and clinical manifestations remains controversial.^[14]^ Some patients have symptoms of peripheral circulation insufficiency, such as amaurosis and syncope. It has been speculated that the right heart is supplied by a branch of the left coronary artery, but its reserve capacity is lower than that of healthy individuals or the left coronary demonstrated atherosclerosis. The patient had chest tightness, which was aggravated in our case.

### 3.4 Manifestation of electrocardiogram

ECG manifestations of patients with RCA absence differ. According to the study of Liu et al, about 60% of patients with RCA absence have ST-T changes on ECG, but half of the patients have obvious lesions in left coronary artery branches.^[12]^ Other patients present with sinus bradycardia, third degree atrioventricular block, or atrial fibrillation. Various supraventricular arrhythmias occur in patients with absence of an RCA. It is presumed that the sinoatrial node and atrioventricular node, which mainly require RCA blood supply, need to rely on the collateral circulation of the left coronary artery or the LCX artery to compensate for the increased blood supply. The long-term overload of blood perfusion by heterogenous arteries causes the decline of arterial reserve function, which places the sinoatrial node and atrioventricular node into a long-term ischemic state, and they are replaced by fibrous adipose tissue after a long time, eventually causing its dysfunction. The ECG in our case revealed sinus rhythm, and I, aVL, and V1–V5 ST segment elevation, which was caused by occlusion of the LAD and the right ventricular branch.

### 3.5 Diagnosis of the absence of the RCA

At present, selective CAG is still the gold standard for the diagnosis of coronary artery variability, but coronary CTA, as a non-invasive means of examination, is widely used in clinical application, with unique advantages.^[15,16]^ Coronary artery malformations often cause great confusion to the operator during angiography, which requires repeated non-standard projection positions. It not only causes a large amount of contrast medium consumption, long operation time, and high risk of complications, but also causes misdiagnosis and missed diagnosis. However, coronary artery CTA has no operational difficulties due to coronary artery malformations and has high diagnostic accuracy. Absence of the RCA often needs to be differentiated from occlusion. Although clinicians can indirectly exclude occlusive lesions based on clinical data, such as symptoms, ECG, and myocardial injury markers, multi-slice spiral CT CAG can easily identify such lesions. Coronary artery CTA can also reflect the anatomical relationship between the variant coronary artery and the atrioventricluar and discover other cardiac malformations. Another advantage of coronary CTA over CAG is that both the left and right coronary artery can be evaluated simultaneously.^[17]^ Combination of these imaging methods yields a more robust assessment of coronary arteries and their neighboring structures.^[18]^

In the present case, the patient was first diagnosed by CAG, and the diagnosis was then confirmed by coronary artery CTA. Because the patient had AMI, we first carried out CAG. During CAG, the RCA was not found, and we observed that the LAD artery sent out the right ventricular branch and the posterior descending branch. We thus considered it to be a case of congenital absence of the RCA with AMI. Subsequently, coronary artery CTA confirmed this diagnosis.

### 3.6 Treatment plan

For patients with congenital absence of the RCA, there are no relevant guidelines for specific treatment options at present. The choice of treatment may include either conservative treatment with anti-platelets, anti-coagulation, lipid-lowering agents, coronary artery dilation, or revascularization therapy, including PCI or CABG.^[19,20]^ In this case, we first used drugs to control the patient's heart failure, and then implanted a stent at the occlusions in the LAD and the right ventricular branch. No symptoms reoccurred during follow-up.

## 4 Conclusion

Congenital absence of the RCA with AMI is a very rare clinical phenomenon, which can lead to death if not handled appropriately. ECG, myocardial necrosis markers, coronary artery CTA, and CAG are helpful for early detection and treatment. Medical treatment and PCI remain the best treatments for this type of disease.

## Acknowledgments

Xiang Tian from Baoding No. 1 Central Hospital of China's Hebei Province is appreciated for his help in proof reading the article.

## Author contributions

**Data curation:** Wei Geng, Xiang Tian.

**Methodology:** Qiang Qi.

**Writing – original draft:** Weichao Liu.

**Writing – review & editing:** Weichao Liu.
